# The Passive Origin of the Institutionalization of Power Inequality in the Meaning/Experience of Womanhood in Igboland

**DOI:** 10.3389/fsoc.2020.00008

**Published:** 2020-02-27

**Authors:** Dominic Ekweariri

**Affiliations:** Department of Philosophy, Bergische Universität, Wuppertal, Germany

**Keywords:** power inequality, women in Igbo land, unintended consequences, passive synthesis, collective intentionality, Husserl, Merleau Ponty

## Abstract

This study, which analyzes the meaning and experience of womanhood in Igbo land, reveals a power inequality, captured by the depiction of women as the property of men. Though most of the intuitions found in post-structuralism might be confirmed in our analysis (e.g., that discourse produces the subjects and that language operates alongside power and social control), my greatest motivation in this essay is different: it proposes that power inequality as evident in the depictions of women and their oppressive subordinating consequences therein are *not consciously intended* by all classes of agents while acting in accordance with normal rules and accepted practice; an aspect that is usually lacking in other accounts of the institutionalization of social realities where dominant discourse, collective intentionality etc., are usually emphasized. Whence the questions: What is then the origin of unequal power distribution among the sexes? And what is the origin of unintended but oppressive images and subordinating depictions of women as the property and unequal of men? Inspired by Jean-Paul Sartre, I make an elaborate use of passive synthesis as developed by Husserl's analysis of perceptual objects and phenomenological perception of time consciousness, and as found in Merleau- Ponty's habituation to respond to these questions.

## Introduction

There appears to be a stark polarity of spheres in ethnological findings concerning Igbo^1^ men and women. Whereas, men are given to the public^2^ spheres, women are given to the private and natural. This distinction determines the way in which the two sexes are *permitted to, enabled by, obligated to* or *restrained from* the public or the private^3^. The findings motivate the questions: What explains the inequality of *enablement and restraint* occupied by both men and women in such a society? Since it seems intrinsic to womanhood in that society to be more restrained but less permitted, we are motivated to ask these questions: “What does it mean to be a woman and what is the experience of being woman in Igbo land?”

To begin with, some ethnologists have pointed out that biological differences between the sexes do not provide a universal basis for socially defining what it means to be “woman” or “man.” They rather suggest that “woman,” like its correlate structure, “man,” is an empirical category and therefore investigable—although in specific contexts of place, time, culture, for according to Moore the “*image, attributes, activities, and appropriate behavior* associated with women are always culturally and historically specific” (Moore, 1988, p. 7; My Italics). The last question posed above is geared toward digging up such *images and representations* of women in Igbo land.

In continental critics of institutionalization, in Marxism, Frankfurt school critical theory, phenomenological existentialism (Fleming, 1989, p. 120), and most importantly in post-structuralism etc., substantial work has been done, offering a critique of institutions where discourses are characterized by an institutionalization of power. Part of this is an intense discrediting of the male-saturated discourses of feminism. Most importantly we also find in these modern theories a deconstruction of the meaning of women. In such cases, such as found in linguistic requirements for institutions, “action theory” and “the phenomenological theory of ‘acts’ espoused by Edmund Husserl, Maurice Merleau-Ponty and George Herbert Mead,” social agents are said to constitute “social reality through language, gesture and all manner of symbolic social sign” (Butler, 1988, p. 519). These claims are substantiated with the claims of feminists such as Simone de Beauvoir who has always held as a central theme the fact that “woman” always carries the burden of a historical meaning through the conventions of an institution, i.e., culture. Her statement “one is not born, but, rather, becomes a woman” shows that what is called gender, with reference to the woman, is just “a performative accomplishment compelled by social sanctions and taboos” (Butler, 1988, p. 520). It can be understood like Merleau-Ponty's reflection in the *Phenomenology of Perception* in which “the body is said to be ‘an historical idea’ rather than ‘a natural species”’ (Butler, 1988, p. 520). In brief, all these mean that the conception of the woman (gender) is a cultural interpretation of the natural fact of her sex (biology). In order words, these institutionally critical philosophical positions say that the term “woman” is a constructed social and cultural reality.

Just as in most postmodern philosophies, especially their variants as found in Foucault, Derrida and Lyotard, there is strong aversion to normative positions grounded in privileged ethical claims because for these the normative positions or privileged discourses can fool us into “conventional meanings and modes of being” (Barett, 2005, p. 1). For instance Lyotard defends the “incredulities toward metanarratives” (Lyotard, 1979/1993, p. XVIV). In line with the question of the meaning of women posed above, most poststructuralists will claim that meaning and identity are rooted in language.^4^ This will make all meanings simply provisional and flexible. It is the society that will determine the regimes of meaning and truth by determining what is acceptable and what is not. Since it is discourse that produces the subjects and since it is language that reveals meaning, discourse will involve an analysis of power.

In our analysis of the meaning and experience of being woman in Igbo land, we shall see how via *constitutive rules* and *collective intentionality*, women are *assigned a function*. In this move *linguistic devices* will play an essential role in the construction of the institutional fact of being woman. Though, most of the intuition found in post-structuralism might be confirmed in our analysis (for instance that discourse produces the subjects and that language operates alongside power^5^ and social control), my motivation in this essay is ***different***: it proposes that power inequality as evident in the depictions of women and their oppressive subordinating consequences therein are *not consciously intended* by all classes of agents while acting in accordance with normal rules and accepted practice; an aspect that is usually lacking in other accounts of the institutionalization of social realities where dominant discourse, collective intentionality etc., are usually emphasized. Besides it argues for the phenomenological account of passive synthesis as capable of responding to the problem of the origin of power inequality and the status function of nwunye/nwanyi (wife/woman).

To achieve this goal, the paper is divided into three main parts: The first part is dedicated to the search for a proper definition/experience of the institutional fact of woman in the specific context of a traditional Igbo society. This part will reveal a structure of power that restrains^6^ women (whether married or unmarried) from certain rights and control over their own lives. Far from just being a mere case of exclusion from public life^7^ and being bound to their domestic and reproductive roles, our analysis of the experience of being woman in Igbo land will expose how the woman is robbed of an individuality of her own from the patriarchal structures that *depict* her as *a property*, thus building up a culture which “damages, destroys, cripples and hinders one from being a human person” (Conrad and Michalik, 1999, p. 438). The second part is concerned with the idea of the institutionalization of power inequality—because the woman is represented as the man's property—which we shall understand as a case of *unintended consequence*. In the last part we shall argue that the *unintended images* of women can be explained by the phenomenological theory of passive synthesis. Note that by unintended, we mean that these images do not come under a conscious reflection in the active sense.

## Institutional Construction of the Meaning of Woman in Traditional IGBO Society

Three elements have been proposed as necessary to account for social realities, viz: *assignment of functions*,^8^ *collective intentionality*^9^ and *constitutive rules*.^10^ The first and the third appear immediately visible in the Igbo social reality to the extent that they prescribe the representation of something other than the natural objects; the second will become evident later. In traditional Igbo society, the fact of being *nne* or *mgbo* (female) *eo ipso* is only negative, as we shall see later. *Being*^11^ is in view of a relationality so that a certain psychology would not ascribe the female her own full, autonomous personality. Instead she requires something more *in order to be*. One grasps here a literal devaluation that is at stake, when one compares the biological feminine (*nne*) with the masculine (*oke*) opposite.

Accordingly mankind/society (*Oha na eze*) is to be seen as a male (*Oke*) so that the man represents simultaneously the positive and the negative poles of a current, just in the same way that the French word *les hommes*, a tag for “man,” simultaneously describes “mankind,” because the specific meaning of “*nwoke*” (*man*—as distinguished from mankind) is absorbed in the generic “*oha na eze”* (man—like in the English *mankind*) the same way the specific meaning of man in the latin “vir” is absorbed in the generic “homo” (mankind); contrariwise the female (*nwanyi*) is insufficient,^12^ whereas “*nwoko*” is (Onukawa, 2000). Accordingly the female's body imprisons and bounds her to herself; the man thinks of his body only in terms of mastery of, and direct relationship to the world. All these make *being* equivalent to man; every other existent is only *on the process of becoming*, and can only be so derived from him, in the same way that the ancients derived geographical orientation from sunrise and sunset. De Beauvoir's insight is *ad rem* to the Igbo situation being addressed: “Mankind is masculine, and the man defines the woman not as such but with reference to himself; she is not seen as an autonomous being” (My Translation^13^). From this basic premise, a second is derived, namely: the feminine is perceived as “a relative being.” Relative to what?

Since just being *nne* is in itself insufficient, she has no place within the traditional socio-cultural framework of the Igbo. In order to overcome this constraint, the *Constitutive Rule* of the form “X counts as Y in C,” as defined in the footnotes above, has to hold sway. In simple language, she who is the X (*nne*) is now given a new status Y (*nwanyi/nwunye*^14^—woman/wife) through a Context (*nwoke*—the man, representative of *oha na eze*) that names something more than what the X as a mere biological condition could afford. Later we shall explain how through the bride price ownership of her is transferred by a group of men to the *di* (master, husband)—a vivid symbolization of collective intentionality seen as an agreement and imposition of Y. In the above process, the body of man is meaningful in itself even without the woman, whereas the later seems wanting in significance, as though the woman *could not think of herself without the man*. In traditional Igbo society, the woman is therefore what the man has made of her. Thus the common parlance, “mma/ugwu nwanyi wu di ye” (Literal translation: “the beauty of the woman is the man, her husband”) (Mmadike, 2014). In the end, one finds it extremely difficult to define the “woman” (now *nwunye* meaning wife) or assign worth to her, when one departs from a specific referential context of the man, and in the institution of marriage. Whereas he is absolute subject, she on the other hand becomes an object belonging to him (his property). This new status function of the woman Y, the property of the man, is designated by the concept wife (*nwunye)*. Finally here, whereas an objective existence is ascribed to the man as such, it is only so by extension to the woman. In order words, a woman cannot be without a man, her foremost substance, if she is to have some place in the *oha na eze*. The renowned Igbo novelist and self-ascribed feminist, Chimamanda Ngozi Adichie painted this traditional psychology in the compelling lines of her novel through Kambili's mother: “A woman with … no husband, what is that? …a husband crowns a woman's life” Adichie (2006, p. 75).

The move from X (*nne*) to Y (*nwanyi/nwunye*) “is *eo ipso* a linguistic move” (Searle, 1995, p. 63). So “language is essentially constitutive of institutional reality” (Searle, 1995, p. 59). This means that institutions like a specific culture must be able to contain linguistic elements—symbolic devices expressing things beyond themselves—of the facts within that very institution. Now the challenge is: what linguistic representations are there to convey the new status function of the *nwunye* (wife) as the man's object or property?

On that count, the Igbo language is rich with such symbolic devices. After she is married, she drops her maiden name and assumes a new name which will be given her by her *di* (husband). We hear of such names: “ure di ya,” “Aku di ya” (“the joy of the husband,” “the wealth of her husband”). In each case her name makes reference to the husband. This way of naming women in Igbo land has almost faded out into extinction, but it is yet preserved in other forms. When she preserves her maiden name after marriage, as is presently common, it is to be distinguished from those of other women. It is her husband's name that specifies what name is being referred to. In Ndiegbelu Umuemeri Ogwu, it is till date a commonly held custom that women are often addressed and/or distinguished from other women by their husbands' names suffixed to theirs. Thus, such designations as *Angela Amodu, Maria Gini, Maria Bonu* are familiar instances (whereas *Amodu* is the husband of *Angela*; *Bonu* and *Gini* show that this *Maria* is the wife of *Bonu* and this other is the wife of *Gini*) (Ekweariri, 2011). A more common practice is to subsume the woman in the man, in the sense of a “one body theory” (Uchem, 2001, p. 116), by which Christianization and Colonization of Africa from “Christian Europe” reinforced the traditional habits. An example here is: “Mazi na Oriaku Uche Anyanwu,” where the woman is only identified *in terms of* the man who is Uche Anyanwu. The subsuming of the woman within the man's identity is not restricted only to “name-calling,” but also stretches into properties. The woman's properties, if at all, not only become those of the man (Ekweariri, 2011), but also children begotten by her outside wedlock.

The pattern of thinking by which the woman belongs to the man (Nwanesi, 2006, p. 26–28) is corroborated in Henderson's linguistic survey of the Igbo words for husband and wife. Accordingly the Igbo word for husband, *di*, means one in a position of authority, command and high degree of responsibility (he exercises headship and lordship over a house), or one possessing a great professionalism in a given area. An example is *Di ji* (yam expert). Muonwe's (2011, p. 13) reading of Herderson makes a further list of words with *di* prefixed to them, such as *di-ogu* (master of wrestling), *diochi* (an expert in palm-wine tapping), *dimkpa* (a very great man). Whereas, all the Igbo linguistic words for *di* (husband) denotes a certain form of lordship/greatness, that of the woman *nwunye* (wife) contains in it a reference to the head of the house, to whom she is subordinate. This is how the requirement of *linguistic devices* is here satisfied, by which speech actions are predominant.

So far we have tried to decipher the meaning of women in traditional Igbo society, with the result that the woman is a relative being, relative to the man without whom she has no identity. The analysis culminated in the idea of the *woman as a property*. This apparent claim has to be pursued further in the four different traditional attitudes below to make clearer the dialectic of unequal power distribution between men and women, in terms of how they are being *enabled, obligated, permitted and authorized*. After we have derived the *theoretical meaning* of woman in Igbo land, it is in the following part that the *experience of being woman* in Igbo land will begin to emerge.

### Preference for Male as Against Female Children

There is little regard for the female in Igbo land when viewed from the optic of an almost idolatrous crave for male children. Such a preference for the male child is functional, as is evident in the connection between death and fertility, by which living men do anything under human power in order to receive a befitting burial, a feat which can be thoroughly achieved by a promising male child. An Igbo proverb contains a male-child's (the first male child—Opara) traditional duty of burying his father: “*Onye aka ruru ya lie nna ya owughi opara gburu”*: literally an appeal to any of the male children of the man to bury their father if they were well to do. The *Opara* must not be waited for since he is not the one that is responsible for the father's death (Onwudufor, 2015, p. 56). The more children a man had, the more he was assured of a proper and befitting funeral at death. A befitting burial is necessary to secure peace in the land of the ancestors and a key to the attainment of the title of an ancestor. Only a male child is authorized to pour libations and offer sacrifices which were believed to placate the dead and keep them at peace. Besides, the ancestral lineage faded into extinction if there was no male child to immortalize his father and continue his name. Thus, the masculine names *Ahamefule* (may my name not be lost), *Obiechile* or *Amaechile* (May my home or compound not go into extinction), *Ahamdi* (My name still exists), are prayerful supplications made to one's *chi* (a personal ‘god’) to spare him the wrath of any extinction. Also is the need to preserve and secure one's ancestral properties, which only male children could realize. Only men could become holders of the *ofo* (the symbol of authority and justice), represent the family in both the spiritual duties of offering sacrifices to the family deities, and perform the very important ritual of the kola nut (Ekweariri, 2011; Iheanacho, 2012).

The begetting of a male child does not only serve the man; the woman's status in the family and society becomes permanent and she herself is filled with joy because of the respect and the status which her male child acquires for her, a status she would never have achieved with the birth of a female child and worst still with barrenness.

The birth of a female child or especially chains of female children was a source of worry for the family; it was like a scourge and curse to the woman and family. Her husband castigated her for giving birth to “good-for-nothing–children.” Besides, the girl child is conditioned to accept subordination as her lot. It begins in the homes where the distribution of household chores is made to favor the male child, and place the girl child as the servant of her brothers. As Nkachukwu lamented, “Boys are allowed to play all day and girls are held down with domestic work” (Uchem, 2001, p. 94). She would later be rewarded by being married (Thus a famous Igbo historian writes: “that when a woman outgrows the question ‘whose daughter is she, people would ask, ‘whose wife is she?”’ (Amadiume, 1987, p. 69) if she imbibed those qualities which men have prescribed for future wives: obedience, respect, servitude and humility. She would be punished by the absence of suitors if she failed to imbibe these qualities. She learns early that the man and children are her goals, that the man is her master and could do literally all he wanted including that of taking other wives. For Okoye, this belittling of women even shows itself in cases of bailing out a prisoner in which “a female corporate manager or top executive is often asked by brash policemen to go and get a man, even if he is the lowest male in the employ, such as her gardener, to perform this function on her behalf” (Okoye, 1995, p. 4) as if she were not human enough.

### The Biological and Domestic Values of Women in Igbo Land

Whereas, the socio-political and cultic spheres in Igbo land are male-saturated, responsibility and service in the man's homes were the exclusive reserve of women. As custodians of the man's home, her primary way of serving it was through child bearing (Entwisle and Coles, 1990, p. 266), and a failure to do so was punishable by social ostracism. Among the Igbo, a woman's primary goal in marriage is to service the ancestral line of the man.

There was no gainsaying the fact how important the child (especially the male child) is to the man in traditional Igbo society. This same importance appears to be a burden for the woman, who lost all respect, unless she proved herself to be fertile. The onus of proof was primarily on the woman. First to be fulfilled as a woman, not only marriage is necessary but also begetting a child for the husband. Again in traditional Igbo society, the child is important to the woman because it secures for her a home and permanent residence within the family.

Having many children implied an increased income for the man since they assisted in farmlands, in trade and the professions of their parents, to bolster the wealth of their families. Whereas, the farmland had men as the CEOs, women and children were sources of labor and manpower (Ekweariri, 2011). It is therefore not surprising that such festivities/rites as *igbu eghu ukwu* (reserved only for women who gave birth to ten children or more) or *ile omugwo* (a special rehabilitation of the woman after childbirth) developed in this culture in order to celebrate fertility. Together with their prerogative of nursing and bringing up the children, Igbo women, as wives and mothers, would be seen as shouldering great responsibility in domestic functions.

### The Bride Price and the Subordination of the Woman

Marriage in Igbo land is not just an isolated affair between two lovers; it involves the whole community, the extended family, the kindred, and the special role of the *ama ala* (the council of elders). It is the second most important celebration after the birth of the child. The symbolism of marriage contained in *mmanya ajuju* (the wine of inquiry), *nleta ala* (home visit by the bride to the groom's house), *nkuzu mmanya* (completion of wine) and the *emume olulu nwayi* (the marriage ceremony *per se*) reach a culminating point in the “bride price” (Iheanacho, 2012).

The “bride price” is a certain amount of wealth (or money etc.,) given to the bride family in exchange of their daughter. It is a concrete expression of gratitude to the family of the bride in compensation for the pains of upbringing and education. It could be seen as that which effectively seals the contract between the family/kinsmen of the bride and that of the bridegroom, and confers the right of ownership to the bridegroom. Women do not usually profit economically from this transaction and transfer of ownership, outside the achievement of a proper social status of “a married woman” (Aina, 1993). Moore writes: “In bride wealth transactions, *groups of men exchange goods for women, or rights in women, between themselves*. Women would appear to profit very little by them as individuals. The rights transferred with the bride wealth valuables involve the transfer of right of children” (Moore, 1988, p. 70). The italicized above shows the satisfaction of *collective intentionality* and it is in this way that “X (*nne*) counts as Y (*nwunye*) in C” (the gathering of elders—men—who transfer right of ownership of the woman using a linguistic formula), a shift which does not go without the institutionalization of power and inequality. Does this then mean that power inequality arises from this *collective intentionality* of a group of elders representing the *oha na eze* since masculinity is the normative dominant discourse? In section The Institutionalization of Power Inequality as Unintentional we shall argue otherwise: that the intentionality of inequality here at play is of another type—inequality is actually *unintended* and therefore springs from a different process.

There are no doubts that the bride price is often exploited, when her kin try to commercialize her qualities such as beauty, intelligence, industry and enterprising spirit, when they try to make gains out of these, and sale her off to the greatest bidder. Otherwise each kindred unit was autonomous in fixing their own bride price and drafting their own list. The family and *ama ala* (this refers to a group of elders who played a major role in the marriage transactions and transfer of ownership) of the groom would go home to work hard to ensure there was nothing left behind in the list so as not to spoil their face in the house of their future in-laws. On the day of the marriage proper, kindred members of the groom will help to deliver the prepared food, drinks and the material things which are part of the bride price to the house of “their to-be wife,” accompanied by dance groups or songs. When the preliminary parts of the traditional wedding must have been over, “the kinsmen of the bride's father would demand to be paid with signs and in secret… Many communities did not even allow the name of money to be mentioned in any transactions regarding marriage… they did not want to give impression of selling their daughter for money… In some communities, there was no stipulated amount to be paid …Families were allowed to pay whatever they could afford” (Iheanacho, 2012, p. 205–206). Even when the transaction of the bride price did not directly imply any economic gain for her, the social weight of the woman was measured according to the fatness of the bride price.

The rituals of the bargaining, compromise and paying of the bride price is beyond women's affairs; they were not allowed to participate as David Iheanacho's documentation shows. With the payment of the bride price, the man exercises a claim to the woman; with it also the man is legitimately entitled to the children born inside and even illegitimately outside of wedlock by the woman. In case of divorce, the man retains an absolute right of control over the children. At the end of the ceremony, the bride follows the groom to his home; she knows that she is no longer her own.

### Social Torture of Unmarried Women

We have analyzed the “woman” in Igbo land as a relative term derived from the man, an anthropological base from which her freedom and independence are measured. If the woman abstains from this base, she is not integrated in the society and suffers severe consequences (Entwisle and Coles, 1990, p. 266). That means the aspirations of the community is imposed on her as a way of life. She is a product elaborated by culture. She cannot but lose her personhood, if not coupled with the man, to become a *nwunye* (wife).

Any female who finds herself in this group is counted as most unfortunate. What fate has left for such is isolation from the community. They live their humanity as though without using their means and without reaching their end. Because they have not been fruitful, they see themselves as failures. In the stratification of the society, no place is reserved for them except the despicable nomenclature: “*Oto n'aka nne ya”* (the one who remained bound to the mother without gaining her own natural fulfillment—marriage). This stigma placed on them is hard to erase as they carry it all through life, wishing they were never born. Even after life, this stigma remains. They are thrown away in evil forests or waste lands for they deserve no memories behind. In the list of venerable ancestors they can hardly secure a minimal position and no one would wish to be like them since they have become a synonym of an accursed life: No suitable place of rest, no suitable place in the list of ancestors, no place in the memory of the succeeding generation (Ekweariri, 2011). To be given into the custody of the man in marriage remained the only way to avert *this burden of being*. Now we can understand the aforementioned claim that share biology (*nne*) is negative in the Igbo social structure and in this way we have attempted to give answer to our initial question: what is the experience of being woman in Igbo land?

## Discussions

### The Institutionalization of Power Inequality as Unintentional

The above considerations have helped to bring out how relations in traditional Igbo society construct women as “property” through the institution of marriage. Within such institutional structures, “women's ability to act as fully operative individuals in relation to property…is always less than that of men” (Moore, 1988, p. 72). In relation to men they are less able to act as full blown subjects who possess an independent existence. One sees here a *dialectic* which confers a great deal of power over women—of ownership, determination and control—to men. Women turn out to be silenced by the dominant structure of power^15^ that makes them the unequal of men, as we have seen in the four attitudes above. This *dialectic* is what we refer to in this paper as the institutionalization of power inequality between the sexes. Some have interpreted this power to be constructed within the dichotomy of ‘male is to female as culture is to nature’, where the first stands for public and the second for domestic. Whereas, the former is preferred to the latter, since it is always the case that culture seeks to transcend and exploit nature, and therefore superior to it and whereas the activities of Igbo men in traditional society are associated with institutions, governance, authority, women's were based on reproduction, nursing, and domestic etc. It is worth questioning why women are tabooed from the public sphere, and places of authority in traditional Igbo society, why a certain patriarchal psychology still treats them as lacking an autonomous existence (they do not belong to themselves), and why a socio-political culture constructed by the oppressive male caste should be sacrosanct. We can compare such an institutionalization of power inequality to the institutionalization of “God”^16^, in any religion, which puts women on the margin of the society.

While setting the man as the foundation of all things the Igbo socio-political structure has succeeded in laying the foundation for all other subordinations that has become an existential burden for the woman. Whether she is treated as property, denied of participation in important decisions of the family/society and in inheritance of family goods, given away in her early years as a maid, sold into marriage with the bride wealth, confined to the home and domestic works (Ekechi, 1995) which made men “bread winners” and women “home-makers” (Steady, 2001, p. 38), forced into early marriage because of accidental pregnancy, not given a fair chance in career as her male counterparts are, tortured for not being married to a man or for being barren, the Igbo woman today stands in a certain disadvantaged position, precisely because power is unequally distributed.

Now if we depict this as a moral wrong (for morality involves the active use of the faculties of reason and freedom: i.e., it involves intentionality), it will appear as though, men in Igbo land were actively thinking out ways of marginalizing their women counterparts. Though the status function and the experience of women were the collective construction of the dominant discourse of masculinity, *we do not think that such a “collective intentionality” did undergo conscious reflection*. If we make this claim, it is precisely because this type of moral wrongness distinguishes itself from all other moral wrongs deriving from a specific conscious intentionality or from the reflected actions and policies of some groups.

The morally wrong image and depiction of women as the unequal and properties of men Igbo land derives neither from her own choices nor from her actions, for it is something beyond her own control and choice. Neither does it originate from the *directly willed choices* of others nor their actions, nor does it derive from any particular unjust law or single unjust policy consciously imposed. This wrongness does not directly relate to her life, but to her *position* of being vulnerable to unjust treatments. Contrariwise, women's vulnerability to unjust treatment is a “multiply, large scale, and relatively long term” (Young, 2011, p. 47) process, deriving from private and public policies “and the actions of thousands of individuals acting to normal rules and accepted practices.” Thus, investigating the origin of the institutionalization^17^ of power inequality has to put into consideration the fact that the multiple processes at play might not be premeditated acts.

The *multiple processes* alluded to above could come in the following forms: (1) “*objective constraints”* (these are those formal and informal **social rules** that tend to hinder her from being justly treated: e.g., that women could not inherit ancestral lands; that the man should protect his wife by walking ahead of her, were they to be on the way; that females were not to appear before the *ama ala*^18^; that women do not and could not bail anyone from prison); (2) “*positions”* (these have nothing to do with her as a person, e.g., as Ngozi, Maria or Ahuruchioke, but as the way in which women stand in relation to others: that she was a 38 years old unmarried business woman, applying to buy a landed property alongside two other male counterparts; that she was a 46 years old barren woman with an edifying career; or a 77 year old grandmother from a large family and who has a lot of respect in her extended family); (3) “*structures produced in action”* (when individuals explore their knowledge of social rules to improve their capacities: a fat bride price, from which the woman could not directly profit, and which in fact authenticates her deprivation of any autonomy would increase her social weight, if she could explore her knowledge of social rules and exploit social relationships to acquire it; since a populated home was advantageous to the man, both in servicing his ancestral lineage, ensuring a befitting burial and securing for him a venerable place among the ancestors, women who were very fertile were treated in certain ways in relation to others: from this predisposition could emerge institutional manners of honoring such women. An example here is *igbu eghu ukwu* which celebrated fertility); (4) “*unintended consequences”* (in this case the woman's exposition and vulnerability to unjust treatments is an outcome of **a**. individuals—*private persons* like siblings, her father or mother,—and **b**. *public institutional policies and practices* such as those of the council of elders or the *ama ala*, who while acting did not foresee these consequences. Examples: Consider in the first instance that the young woman's father promised to marry her into a noble family, because she was respectful, self-effacing, obedient and submissive to him. The father does this purely out of good will and no wrong was ever intended. The young woman rejoices because she is on the right side of series of events that would lead to her socio-cultural actualization. Consider again in the second instance that is the prerogative of the *ama ala* to carry out the marriage enquiry, *mmanya ajuju*, and whether her being uplifted to the next social status of marriage would take place, depend, to a large extent, on their recommendations and consents. Note that the council of elder's decision to do this is purely based on the “good-will” of protecting their “daughter” from any unhappy wedlock. Thus their duty is to investigate the bride groom's family background and to ascertain that it was free from any history relating to certain mental diseases like schizophrenia or that the said family was neither cultically impure as an *osu*—an outcast—(Odunze, 2017), impeded from marrying their daughter who is a free-born or *nwadiala*, nor unfair to women. However, all these might result in a subordinating depiction of women).

As we have seen the *multiple processes* are internal to the dynamics of that given society and do not derive from any single intentionality, action, policy or practice. All these work hand in hand to produce effects that were neither intended nor consciously premeditated in any way, and in most cases beyond any specific institutional fact. While carrying out their cultural assignments, the individuals in the council of elders were not conscious of selling their daughter; neither were they aware of how they were making women their unequal by institutionalizing subordination. If at all, they would be considering themselves as carrying out honorable acts by keeping the traditions handed over to them alive; more so they would be happy for “their” daughters who were actualizing their potentials by being married and begetting children, and be worried about those who were not.

If the status function of *nwunye (wife)* was connected with power control by the dominant class, as the four traditional attitudes above highlighted, since it made women vulnerable to unjust treatments (subordination, oppression, ostracism) in relation to their male counterparts in Igboland, it becomes expedient to inquire from where these unequal *deontic powers*^19^ originate? If it does not originate from a collective intentionality, does it mean that it originates from a non-intentionality with regard to institutional facts? For instance, the *intended* function of money is to be a means of exchange; its *unintended function* could be to maintain the system of power relationship in society. In like manner, among the Igbo the *intended function* of the institution of marriage is the mutual protection of the rights and social interests of the parties, but the *unintended function* might imply the *enablement* of ‘some’ (the institutional fact of *di* exercises a constraint on the institutional fact of nwanyi/nwunye) and the *subjugation* of “others” (in the case of *nwunye*, social status is to be recognized by their power of external coercion on her;^20^ the woman is represented as the property of the man).

It is this *unintentional* aspect that is usually lacking in accounts of the institutionalization of social realities where, in its place, dominant discourse, collective intentionality (that is the case of someone or a group of people thinking it so, (Schweder, 2007, p. 97/98) are usually emphasized. Though scarcely accounted for, the unintentional aspects play a serious role in the subject's constitution of the world or of social realities. It is therefore of a great importance to us, to highlight the origin of such unintended but subordinating images and actions, which are characteristic of the experience of women in Igbo land.

## Explaining the Unintended Images and Depictions of Women via Passive Synthesis

Our motivation in seeking for answer to the origin of unintended but unjust actions (oppression, subordination) stems from Jean-Paul Sartre who expressively wrote in his *Critique of Dialectical Reason*:

These considerations are not intended to make oppression *into the direct, historical origin of class division and exploitation*. Far from it. On the contrary, we recognize - because it is obvious - that the practico-inert field of exploitation constitutes itself, through counter-finalities and through the meation of worked matter, as *a passive synthesis of serial relations”* (Sartre, 2004, p. 737; Italics are mine).

Continuing Sartre writes:

Whether we are dealing with slavery as an institution or with the consequences of the division of labor, it is impossible to treat the material, technical, demographic, etc., development of a given society as *the objectification of the free praxis of an individual or group*. It is undoubtedly true that - as Engels says - slaves appear at the moment when the development of the techniques of agriculture makes them possible and necessary, that is to say, that *an institution is a response to the practico-inert exigency of an already constituted field of passive activity* (Sartre, 2004, p. 737–738; Italics are mine).

In other words, what Sartre is saying is that the division of the society into class—in our situation the division articulated between the dominant masculinity and the “inferior” femininity or in the collective intentionality of a dominant group of elders—is not the place to look for, when searching for the origin of power inequality or of oppression. Neither does the constitution of social realities (e.g., oppression) emerge from the directly intended goals of individuals. Though it is clear that the type of oppression he was referring to in this text, inspired by a reading of Engels, is associated with the condition of workers in a capitalist system—which constitutes some as slaves or workers and others as masters or employers—yet Sartre is clear about this: oppression neither springs from such divisions nor from the conscious subjective decision of individuals (he called it *the free praxis of individual or group*). Contrariwise oppression emerges from the internal constitutions and dynamics of a system (which he termed *the practico-inert exigency)*, which constitutes itself in a serial order, the affectation of the one being the affectation of the other, and the affectation of the other being that of another etc., without any of these being consciously intended (multiple process). That means that institutions (like oppression, inequality, classes etc.) arise from already available passive structures. Institutions produce themselves pre-reflexively; they spring from a passive synthesis. Sartre is not alone in this thinking. Elsewhere the production of racism and oppression in general has been respectively accounted for by passive synthesis (De Roo, 2013; Nethery, 2018).

It was however Husserl who in *Aktive Synthesis*: *Aus der Vorlesung “Transzendentale Logik” (1920/21) Ergänzungsband zu “Analysen zur passive Synthesis”—*where the aspect of “activity” in the title simply indicates how subjects constitute their world and the aspect of “passivity” indicating how these same subjects are being constituted^21^—developed the phenomenological theory of *active* and *passive synthesis*. For the sake of clarity, in the aspect of activity, the “I” generates and constitutes new objects as products. The examples which Husserl himself gives show the activity of reason, when it performs “division” (thus constituting parts: “Imo state has 27 local government areas”), “counting” (constituting numbers: “Anne's Apartment has 6 rooms”), “predicating” (making predicative judgments) etc. (Husserl, 2012, p. 80). Accordingly, it is in the acts of the “I” (*Ichakte*) that the consciousness of the objects (Gegenstandsbewusstsein) is accomplished. Even in all experiences without objects (such as in art works, economic goods), a higher level of objectification is operative whenever the “I consciousness” performs objective value predicates (Husserl, 1920/21, p. 7): “Sandro Botticelli's *The Birth of Venus* is a classical masterpiece”; “In the 1950s Germany experienced a *Wirtschaftswunder*” (economic miracle). We can see that this dimension is explicit. However, as soon as^22^ these explicit reflective performances are made by conscious act of the ego, they “sink again into passivity” (Husserl, 1920/21, p. 5) and become for future use implicit. How does this happen? To account for this we now turn to the aspect of passivity elaborated by “passive synthesis.”

Passive synthesis captures those dimensions where our experience of things is pre-given, (*vorgegeben*), in advance, and already accomplished. For Husserl, what encounters us in life, so to say as just ready, completed stuffs—say hammer, table, etc.—is given in the originality of “itself” in the synthesis of passive experience (Husserl, 2012, p. 80). Consciousness can in active reflection always fall back to these “pre-determined” experiences.^23^ For instance when I am trying to perform an objective value judgment about this piece of art work before me, I fall back to already pre-given and finalized structures such as internalized vocabularies that are not currently being reflected over: “masterpiece,” “classical,” “Botticelli.” Thus: “Botticelli's work is a classical masterpiece.” This means that pre-determined' experiences are evoked in each active synthesis. However, it does not mean that the predetermined experiences underwent a conscious (explicit) reflection since they are automatically evoked. But how was this predetermined experience constituted in what Husserl named primary constitution (*Urkonstitution*)? Husserls response to this can be found in the analysis of perceptual objects and in the phenomenological perception of time consciousness. In the analysis that follows I take the liberty to merge these two aspects together.

In the “Analysis Concerning Passive Synthesis,” Husserl identified a contradictory aspect of external perception which consists in the limitation of the processes of appearing (*Erscheinungsabläufe*) and “beyondness.” When we perceive spatial objects they appear to us in a limited fashion in the ***present***. We see *only one* side of them. Yet they “present” to us a plurality of appearances (possible perspectives and sides of appearing) which are not immediately perceptible, “a beyondness” (Husserl, 1966, p. 3–4). This seeming contradiction, or tension, between the “already perceived” (fullness) and the “not yet perceived sides” (empty) is rather a continuum, for the former points to a “co-constituted” “not yet,” and the latter makes the “already perceived” possible in the first place (it marks its openness). The emptiness of the “not yet perceived sides” does not imply that the “not yet” are “nothing”. Rather it is an emptiness with intentionality, or a “determinable indetermination,” once we go round the object and behold the other side. In order words, the intentionality of the “not yet perceived” can become fulfilled. When this happens, the process is repeated in each phase of perception, so that new empty horizons of possible appearances merge into fulfilled appearances. This process, which Husserl termed adumbration (*Abschattung*), of external perception captures the flow of experience which is given in time. This implies the merging of the “already perceived” into the “not yet perceived” (of fullness into emptiness), in which each advancing fulfillment of empty intentions corresponds to an emptying. In simple terms, immediately the world or perceptual objects are experienced they increase my knowledge (perceptual experience); they become determined and no longer determinable. Thus empty, though fulfilled. But this knowledge will disappear, or experience in general will not be possible, if it were not held in place (Ekweariri, in press). We would only have single rapidly flashing points of linear appearances that are not connected to one another.

It is ***retention*** (capturing the past), together with protention^24^ that secures an “acquired” experience for a future use, for experience cannot exist outside these dimensions of time. An acquired sedimented experience (this is possible thanks to the retention) can be *relieved* (*Vergegenwärtigung*)—for Husserl it is freely available (*frei verfügbar*) and makes the world already always given to us in advance—each time. Experiences that have fallen into retention will lose their specifications with time and will take on a general view which can be re-awakened via *association* in future situations, when one comes to similar situations. For Husserl, association involves an affective awakening of a past experience which is connected by similarity (Ä*hnlichkeitsverbindung*) with present experience. It is a “purely immanent connection of ‘this recalls that,’ ‘one calls attention to the other”’ (Husserl, 1985, p. 78). This process occurs passively^25^ without my conscious intervention. You come into your working room and go directly to the writing desk. This is automatic because the presence of the chair exercises an affective pull linking your past experience of your working room with sitting and your present condition of being exposed to the same experience. To be more technical, assume you were in Igbo land and you saw a woman walking with her husband on the way. She walks twenty meters ahead in front of him. Immediately you are shocked. Now all your experiences of “couples walking on the street” and a woman walking in front of her husband have been retentionally sedimented in a general sense. One of such experiences could be your mother's commentary that such a woman was not cultured. These experiences are now “awakened” the moment you observed this woman walking on the street. Immediately you saw this woman walking in front of her husband, you were upset. You had no time to reflect what might be the reason for her doing so. All active reflections were bypassed. We see that although an experience might not be active in your field of consciousness, it was however ***passively*** there in an implicit form. Since the experiences in retention are the basis of possible future experience, we can see that it is in the exposition to the *multiple processes/experiences of the depiction of women in a particular way* (as properties of men in Igbo land, as unequal of but subordinate to men) that constitute the sedimentations and not just *collective intentionality* of a class of agents (e.g., *ama ala*) or individuals acting in accordance with accepted practice.

At this point, it is important to note that this structure of perceptual experience of the world (or objects) cannot be complete if mention is not made of ***protention*** (like future expectation). This concerns the “not yet perceived parts,” which are however co-constituted in perception. When the “not yet perceived” side of the object is perceived then the expected perception is fulfilled and take the dimension of expectation with reference to a future possibility, which can be further fulfilled or not.^26^ The protentional dimension is therefore the dimension of *possibilities* because it makes consciousness open to *new possibilities* (Nethery, 2018, p. 287). The protention of past passively sedimented experiences of the meaning of—the images which are pre-given about—woman in Igbo land is activated whenever one comes in cross with a woman in Igbo land, causing one to *expect* this woman to conform to predetermined representations of subordination. With regard to the woman you saw walking twenty meters in front of her husband, you might even unconsciously start expecting the woman to be disrespectful, uncultured, in case you have another opportunity of meeting her in future. A well-known slogan used in daily interaction such as: “when a woman outgrows the question ‘whose daughter is she, people would ask, ‘whose wife is she’?” documents one of the sedimented representations of women. This might predispose men and women to evaluate women's self-accomplishment (success) in terms of a life with a man, and failure in terms of a life devoid of a man. It is from such passive predispositions to possible depictions and representations of women that future power inequalities are exercised.

Before we conclude, we want to highlight this passive aspect of sedimented experience as it is very pointedly developed in Merleau Ponty's *habituation, sedimentations and structures*, which were all articulated with the theoretical framework of the body, to which we shall now briefly turn. We shall only explain the first. To this regard, Merleau Ponty portrays the anonymous pre-personal existence of the body,^27^ where it plays a key role in our making meaning of the world. If Merleau Ponty speaks of the body here, we have to understand it as a general theoretical intuition (of experience) through which individuals make sense of the world. This type of meaning is not set up as reflective thoughts because here the functioning of the body follows a stable and habitual path as evident in the mastery which the typing hand has of the keys: “To know how to type is not then to know the place of each letter among the keys […] It is a knowledge in the hands, which is forthcoming only when bodily effort is made, and cannot be formulated in detachment from that effort” (Merleau Ponty, 1945/1962, p. 143–144).

The way the subject who learns to type, learns to habitually incorporate the key-bank space into his body space, so also is the individual able to incorporate the world in his *data bank* of experience (the idea of the body schema). It is through this means that the body is able to know the world in a non-premeditated but habitual way. Merleau Ponty writes: “habits express our power of dilating our being in-the-world” (Merleau Ponty, 1945/1962, p. 143–144). Habits provide the body with a knowledge that is automatic, like the “reproduction” (or association of past sedimented experience in the context of a present experience) found in Husserl, because the body does not need to consider or reflect about the course and path of action.

The concept of habits appears also alongside with that of “structures” in Merleau Ponty. Structures refer to bodily, social, psychological, cultural and psychomotor formations through which meaning is deciphered in the experience (and orientation to) of the world. So they help us to communicate with and comprehend the world—this involves internalizing the world of reality in us in a given way—and from hence we can relate to the world in a habitual way so that in experiencing we do not need to repeat a long process each time, wherein the familiar is encountered by way of habitually sedimented structures. When structures are transformed and sedimented, they become a part of a habitual way of being in the world, thus expanding and further articulating our experiences (Bullington, 2013, p. 33–34). These experiences are accumulated in our data bank of experiences. Consider *language* and *culture* as such sedimentations of experiences in body schema that predispose in a particular way. Merleau Ponty's idea of the habituation of the body can therefore find expression in the following cultural habits which predispose in advance of all conscious reflection: The *ama ala* or council elders have the sole prerogative of deciding and recommending the suitor to their daughter (each female born into that clan). This tradition might predispose younger women to behave in certain ways in relation to all the male members of a community. Perhaps some members of this council could be predisposed, to exploit these situations as opportunities to increase and develop their capacities, thereby propagating power inequality. Even the *bride price* as a means of increasing one's social weight or the economic and cultic advantage of women—example the *igbu eghu ukwu*—who help to service the ancestral lineage of the man, could predispose both men and women to behave or relate in certain ways that promote power inequality. From most of these multiple processes taken together, individuals in this Igbo society would not be conscious of the way they perpetuate a given oppressive image (representation like the idea of women as properties) of the woman, an image which might predispose them to being vulnerable to be treated as the unequal of men.

## Conclusions

I have shown that the phenomenological theory of passive synthesis is capable of explaining not only the *origin of inequality* and the constitution of *nwunye*/*nwanyi (*wife/woman) as property; but also the origin of all *unintended but oppressive images and subordinating* depictions of women in relation to their male counterparts. By *unequal* we have referred to those positions of subordination that being *nwanyi or nwunye* would entail in relationship to all other agents in the socio-cultural milieu of the Igbo. More so, whereas collective imposition of status function using constitutive rules—that is linguistic devices—sufficed to explain the meaning of women in relationship to the man, I have shown that this same criterion, while open to *unintended consequences*, cannot explain how these same consequences came about. We think that such is not well-equipped to articulate those problems concerning the *experience* (oppression) of women in Igbo land.

The path to overcoming such *unjust images of, and action toward women* needs to acknowledge this origin of all *passive and unintended, but unjust images and actions*, since it would contribute to the first steps in the bid of changing the social relationship between the socially constructed categories of “woman,” “wife” and “man,” “husband” in Igbo land. The passive origin can also explain why inequality seems even to persist over time, even when the contexts of their original constitution have totally disappeared. For instance, even though the traditional habits such as *igbu eghu ukwu* as mentioned above are no longer ubiquitous and popularly celebrated, yet barren women continue to solely bear the burden of childlessness, in comparison with their male partners. And though for present generation of adults it is for the most part unobtrusive when the traditional habit of women walking behind their husband are no longer observed, women continue to expect their future partners to be protective bread winners of the family who take the lead in all matters such as decision making etc., while men continue to expect that their future wives be totally subordinate.

Though some of the original contexts of some of the “primal constitutions” of passivity (*Urkonstitution* to use the vocabulary of Husserl) are no longer available, the subordinate image and depiction of women which they carry as content continue to be propagated and produced daily—in some new forms. The Igbo society is still inundated with such depiction of women as men's properties, as subordinate. It is this present general and more diffuse depiction of women that is most powerful since it unstoppably strengthens and perpetuates the gap of inequality.

To further corroborate our claims about the passive origin of inequality and subordination, some women or men who travel outside of Igbo land to, say, western societies, experience a sudden radical change in their perceptual experiences. This is because they are now conscious of this field of perception that was implicitly present and could, in most cases, reflect on their cultural habits for the first time.

## Author Contributions

The author confirms being the sole contributor of this work and has approved it for publication.

### Conflict of Interest

The author declares that the research was conducted in the absence of any commercial or financial relationships that could be construed as a potential conflict of interest.

## Footnotes

^1^The Igbo are located in the South—East of Nigeria, West Africa extending to parts of mid-western and delta regions of Nigeria. Their territory cuts across the Equatorial forest in the South and the Savanna in the North, and both east and west of the Niger River, which is a very important physical mark in this regard. The east of Igbo land includes the Cross River Igbo (Edda, Abam, and Aro) and the west embraces parts of Aboh, Asaba, and Agbor in the former Bendel state of Nigeria. According to Ihuagwu's (2011, p. 58) use of the 2006 Census Estimate, “Igbo land has over 30 million people with a density ranging from 1,000 per sq. mile in high density areas and 350 per sq. mile in low density. It could be the densest area in Africa after the Nile valley.”

^2^In socio-political milieu of the sub-lineage or extended family, the lineage-assembly, and the village group assembly/council, men occupy an outstanding representative position (they bear the symbol of authority and justice, the *ofo*, handed down by the ancestors) (Falola, 2005, p. 161). On another note, Igbo traditional religion is predominantly saturated with masculinity. Its concept of God, *Chukwu* or *Chineke* figures a dominant androgynous “God.” The qualities of *Chukwu* and *Chineke* portray the cultural cliché of a sole generating and providential prerogative of an Igbo “father.” Of all the numerous local deities and personal spirits in the Igbo religion and cosmology only a scanty few are female, among which are *ala*, the water goddesses or deities such as *agbara mmiri* and *umu mmiri* and in some literatures *mami wota*. Apart from these few deities which are often associated with either fertility or some negative forces, the others, such as *Ahianjoku, Amadioha, Agwu Ishi, Ofo, Ikenga, Agbara, Arusi*, etc., are vested with a certain dignifying nobility and authoritative finality in their actions and dealings with humans. To experience these forces is nothing less than experiencing the “self-sufficiency” and “assertiveness” of a typical Igbo man. The ancestors who occupy a very important place in Igbo religion are supposed to include every dead member of the family. But only the masculine ancestors are emphasized and highly revered. In the pouring out of libations one hardly hears the mention of a female ancestor, except in the general coinage: “ndi *ichie* bia were nu oji ….ndi *ichie* bia nuru nu mmanyi” and so on, which after all far from being all inclusive is an appeal: “*Oh ancestors come and take cola nut… Oh ancestors come and drink wine*.” In most parts of Igbo land the word *Ichie* (where this refers to a title) is used exclusively for the male. Most African traditional religions include women in their priesthood; but among the Igbo a female priest is hardly mentioned. It is the prerogative of the male. The *dibia* (native doctors) and the *ohu uzo* (seers) are predominantly male. They stand for all (Iheanacho, 2012).

^3^This distinction, not of class, essentially dates back not only to Marxism as pointed out by some feminists such as Mary O'Brien and Allison M. Jaggar (who suspected Marx was himself guilty of the liberal theory which he had tried to displace: “The suggestion that Marxism, in its characteristic separation of public and private life, shares important features of the liberal theory it has sought to dislodge has also been suggested by other feminists” Fleming, 1989, p. 121), but also to the social and political structures that developed in the West in the sixteenth and seventeenth centuries (Fleming, 1989, p. 121). Benhabib(1986, p. 405) has described this sort of normative categorization of men and women in terms of public and private as an oppressive structure. For her it is “an essential way in which social reality is organized, symbolically divided, and lived through experientially.” In *Feminism as Critique* (Benhabib and Cornell, 1987, p. 32), Benhabib and Cornell make direct reference to Jürgen Habermas as one of the best analysts of the institutional division between “system and lifeworld” useful to feminists, where the spheres of public and private are well contextualised.

^4^Poststructural theorizing focuses “on how language works, in whose and what interests, on what cultural sites and why” (Kelly, 1997, p. 19). It is discourse that produces subjects; it is language that reveals meaning. This will make discourse an analysis of power.

^5^Carrie Paechter in her essay “Using Post-structural Ideas in Gender Theory Research” shows the categories of masculinity and femininity are saturated with *power* and knowledge relations and that they act upon individuals in particular societies. She writes: “What this means is that how individual males and females behave involves an enactment of their gender roles, as interpreted by them in their social context and as constrained by their experiences of their bodies. What we need then to consider is *how discourses of masculinity and femininity are constructed and maintained*, and how they support and are supported by power/knowledge relations” (Paechter, 2001, p. 6 My Italics).

^6^This idea is found in Searle (2007, p. 21) who had confessed: “In The Construction of Social Reality, I pointed out that the main purpose of creating institutional facts is to increase human power, and that the types of power in question are deontic powers and that these bottom out in individuals”. Elsewhere he (Searle, 2010, p. 91) sees these deontic powers as the truest test of whether a phenomenon is an institution or not, and for him these powers include, rights, duties, requirements and authorizations.

^7^It is fascinating how democratic growth brings with it equal rights for all, which are not yet found in the traditional society. Despite traditional restriction and stereotypes, it increasingly tends to offer women and their male counterparts equal opportunities, such as in education. Contrariwise the gaps of inequality are still wide: women are almost tabooed out of the public spheres in the traditional Igbo society, but bound to maternity and a domestic life, which is, for us, a means of control, restraint from the dominant group that construct them.

^8^Here a core human ability of imposing functions on natural objects with a view to performing the specific functions assigned to them is recognized. These functions are never intrinsic to the objects but are extrinsically assigned, thus observer- related, by which is meant according to the purpose and interests of the users—“agentive”; they add a vocabulary of values to their objects—the normative component. In some instances it could be that the agentive functions assigned to objects are that of representing something else (Searle, 1995, p. 21) or something other than themselves (symbolism). The functions assigned could be intended or manifest. An example is a piece of paper assigned the function of “euro” (money).

^9^The second important feature is the term “Collective Intentionality” which signals the capacity for cooperative behaviour, in addition to sharing states such as beliefs, desires, and intentions. An example of a shared intentionality will be that I (as a pianist in an orchestra) play my part in our performance of the symphony; this example is obviously different from an accidental synchronization of parts. The reductionism of ‘We-intentionality’ to an “I-intentionality” in a bid to mark the difference between singular and collective intentionality is untenable. This reductionism is unable to sum up to a sense of collectivity which itself is “a sense of doing (wanting, believing, etc.) something together; and the individual intentionality that each person has is derived from the collective intentionality that they share” (Searle, 1995, p. 23/25). An example is that representatives of the EU countries met to agree that this piece of paper should serve as Euro notes (money).

^10^*Constitutive rules* can be distinguished from *regulative rules*. *Regulative rules* (like that of driving on the right—hand side of the road) merely regulates; but constitutive rules create the possibility of certain activities having the form “X counts as Y in context C,” (where X names the physical features of the object; Y names something more than this physical object; C depicts the context). Institutional facts (e.g., property, woman, money, husband, or wife) exist within systems of these constitutive rules, and are constituted by application of the formula above. For instance, performing such and such speech acts on this man and this woman standing here (the X term) in front of a presiding official (the C term) the two now counts as married (the Y term).

^11^One is not just something; one is someone only in relationships. For a man, being such is sufficient for being someone. It is not same for a female. Some proverbs would drive this home, such as: “Nwoke kuru nwanyi ihe agaghi isi okuru mmadu ihe” (literally translated a man that beats up a woman has beaten up no one—no human person). More so, the differences evident in gender-based names make this sort of discrimination, as documented in “Okeeke” and “Okaafo” (Male specific); “Mgbeke” and “Mgbeafor” (female specific) document this. On closer scrutiny it becomes evident that males were named in association with great deities like *Ala* (earth deity), *Anyanwu* (sun deity), concepts of greatness like *Duru* (Great man of nobility), or nobility like *eze* (king), revered titles like *Nze na ozo*, religious objects *ofo, Ogu* (symbol of authority, innocence, justice), in terms of social entity like *mba* (a people), ibe (fellow), whereas, women were named in association with dainty issues, virtuous qualities such as Mma (beauty), Akudiya (wealth of her husband), Anuri (Happiness), Ola (precious ornament), Ure (conduct worthy of emulation), etc. (Onukawa, 2000, p. 107–108) These system of naming women seem to suggest that women are being instrumentalized for other purposes.

^12^Onukawa's survey of Igbo Gender Naming implicitly pointed out this difference, whereby women were named with reference to a sort of derivable personal and small god (*chi*) and men according to a sovereign, self-sufficient and great God (*Chukwu*). We abstain from the details (Onukawa, 2000).

^13^“Die Menschheit ist männlich, und der Mann definiert die Frau nicht als solche, sondern im Vergleich zu sich selbst: sie wird nicht als autonomes Wesen angesehen.” De Beauvoir (1951, p. 12).

^14^Both terms make meaning only in a relationality. Nwanyi literally meaning “our child” (where our: anyi and child: nwa) whereas Nwunye follows almost always a possessive adjective: nwuye m (my wife); nwunye anyi (our wife).

^15^These are those structures we have examined in the four major features above by which socialisation, right from early childhood, inculcates a set of attitudes by which power relationships take root in social structure. Part of this pattern is that women become a muted group whereas men become enabled groups. Henceforth, we see this power as that which introduces inequality.

^16^In this purview, Conrad A and Michalik K sarcastically probed the male caste for erecting an oppressive image of God that is disgusting to Western women: “why do men worship a God whose major quality is power, whose interest is to subjugate others, whose fear is that others could be equal and become somewhat like him; a being that is addressed as Lord or to whom power is insufficient that his theologians have to even certify him omnipotent. Why has this symbolism become pronounced, and why was that so important for those who thought over it? What fantasies are behind it? Does it amount to saying too much if I name these fantasies phallocratic, namely the worshiping of power? Why should we as women worship and love a being who does not transcend but stabilizes the moral level of the current culture determined by men? I have asked myself how I could wish power as a central category of my life” (My Translation). In the end they claim that to identify with such an aggressor, dictator and violator would be the worst mishap that can happen to a woman. If Conrad A and Michalik k were sarcastically mocking “God,” it is because such a ‘God’ was not actually a God, since he was rather fearful. But fearful of what? Indeed this “God” is referring to men who have assumed his place, and erecting the image of God that will forever disqualify women from being their equals. It is exactly in such a move that the whole institutionalisation of power was based on the institutionalisation of inequality. In the end Conrad and Michalik pleaded for a “God” who gives women the chance to be humans.

^17^We are adapting Young's idea of *structural injustice* to describe this same institutionalisation which tends to make women vulnerable to an unjust treatment without any of the agents directly willing nor being responsible for it. In most cases other subjects who do not find themselves in the Igbo woman's situation tend to benefit from it. Whereas women who find themselves in this group are exposed to such vulnerable and unjust treatments (exposure to ostracism, subordination and oppression), they suffer these situations as individuals and are unable to negotiate power with the massive institutional structures in place; they are deprived of the means of developing and exercising their capacities (Young, 2011, p. 52).

^18^The *ama ala is* also a public forum where all socio-political issues are discussed. The Igbo traditional society prohibits women from partaking in such gatherings. The only exception to this rule can be secured through invitation. In most regions of the Igbo society, the *ama ala* took place during the times evening meals where being prepared by women. Once the men are back from such gatherings, their meals were already ready.

^19^The fact here is that the Y (this has been explained above) term, through collective recognition creates new powers, e.g. *authorization, permission, enablement* etc. Through a collective agreement, a sea of possibilities is opened, so that one can do things one would otherwise never have been capable of previously doing (Positive). This is the first aspect of the deontic powers; in the second, the agent is *required, obligated, compelled* to do something he would never otherwise have done (negative). These powers are a matter of convention, and they are meant to regulate the relationship between people.

^20^The individuals are women who are denied one (Durkheim, 1938, p. 10). This passage is cited by Searle himself.

^21^In Freud (2018) we might speak of the realm of consciousness and the realm of unconsciousness as corresponding terminologies. The symbolic that is unleashed during dreams shows that, though the dreaming subject was on the realm of unconsciousness, there is a condensed meaning of the world for the subject, who is not directly participating in it. This meaning is implicitly given as a pre-reflective consciousness.

^22^This as “soon as” of the reflective performances of the conscious ego was named by Husserl as the primal constitution (Urkonstitution).

^23^We cite elaborately Johanna Oksala in order to highlight this point: “Dan Zahavi argues that in Husserl's philosophy the intentional activity of the subject is founded upon and conditioned by an obscure and blind passivity, by drives and associations. He notes that Husserl famously declares, in Analysen zur passiven Synthesis, that his investigation of the problem of passivity could well carry the title “a phenomenology of the unconscious.” For Husserl, the reflecting consciousness can thus never be totally trans-parent to itself. There are constitutive processes of an anonymous and involuntary nature taking place in the underground or depth dimension of subjectivity which can only be uncovered through an elaborate archeological effort.” (Oksala, 2004, p. 17).

^24^The retentional and protentional extension of absolute and pure now is necessary since they make consciousness thinkable/possible. There would not be a consciousness were there no determination of time which transcends the actual moment of now. For instance we would not be able to have a melody which flows in time continuum, but single jerky sounds, were there no retention and protention at the boader phase of the Present.

^25^It is of great importance to point that both the retention (still having grip on the already gone) and the protention are passive functions of time.

^26^This is captured by Gallagher (2011, p. 423): “If I am listening to a favourite melody, there is some sense of what is to come, a primal expectation of the notes to follow, and the best indication of this is that if someone hits the wrong note, I am surprised or disappointed. If a person fails to complete a sentence, I experience a sense of incompleteness. This kind of perceptual disappointment is based on a lack of fulfilment of protention: what happens fails to match my anticipation.”

^27^When we read from Merleau Ponty: “The flesh of the world, (‘the qualia’) is inseparability of this perceptible being that I am, and of all the rest which perceives in me, an inseparability of pleasure and reality” (French version: “La chair du monde, (le ‘quale’) est indivision de cet Être sen-sible que je suis, et de tout le reste qui se sent en moi, indivision plaisir réalité” (Merleau Ponty, 1964), p. 303) Richir would understands therein a usurpation of the theoretical intuition of consciousness by the introduction of this new notion of ≪ chair ≫. Accordingly, “The ‘flesh’ is another, new way altogether of apprehending the reflexivity classically ascribed to consciousness” (French version: “La ‘chair’ est donc une autre manière, toute nouvelle, d'appréhender la réflexivité classiquement mise sur le compte de la conscience” (Richir, 2000, p. 295). For Richir the reflexivity here is equally the same reflexivity in an intersubjective encounter, which now Merleau Ponty generalises. Rather than hypostasizing in the mind, thought or consciousness, Leib (understood too as body) is a place of passage “ein Ort des Durchgangs” (Richir, 2000, p. 295). We now see the body's interchangeability with the mind, consciousness, through which sensation passes across in order to return to itself. Is this not then a sort of opening of the world? It is because of this reflexivity of the body in terms of consciousness of the world that we understand the body in Merleau Ponty as a theoretical intuition by which individuals make sense of the world.
